# Treatment of Ulcers

**Published:** 1883-04

**Authors:** 


Treatment of Ulcers.—Dr. Wilson, in "Notes on the Treatment of Ulcers," remarks that the application of a specially prepared sand to granulating sores has been tried for some time with success, and that it possesses the advantage, since it absorbs the discharge, of seldom requiring removal, so that healing can proceed without interruption. This sand is prepared as follows: It is first heated to a temperature capable of destroying all organic particles. It is then soaked in a solution of one part of bichloride of mercury in 1,000 parts of water. After this the mixture is placed in bottles, and can be used when required. This mode of treating ulcers is not new, the sandy earth of the termite ants having long been used for this purpose by the natives of the West Coast of Africa. This substance was some time since imported by Mr. T. Christy, under the name of "termite earth," for trial in this country, but whether it possesses any antiseptic properties derived from the white ants is not known.—*Scientific American.*

